# Knowledge revision processes during reading: How pictures influence the activation of outdated information

**DOI:** 10.3758/s13421-024-01586-9

**Published:** 2024-06-03

**Authors:** Pauline Frick, Panayiota Kendeou, Anne Schüler

**Affiliations:** 1 Leibniz-Institut für Wissensmedien, Schleichstraße 6, 72076 Tuebingen, Germany; 2 Department of Educational Psychology, University of Minnesota, Minneapolis, MN USA

**Keywords:** Knowledge revision, Reading processes, KReC framework, Multimedia learning

## Abstract

Outdated information (i.e., information that is not or no longer accurate) continues to be automatically activated during reading and can hinder learning processes. Thus, it is important to understand which factors influence the activation of outdated information and, therefore, knowledge revision processes. In three online experiments, we investigated how illustrating updated or outdated information via pictures influences the activation of outdated information. In Experiments 1 (*N* = 421) and 2 (*N* = 422), we varied whether participants read texts containing outdated information that was later updated (outdated text) or texts containing only updated information (consistent text). In addition, the updated information was or was not illustrated by a picture. In Experiment 3 (*N* = 441), participants read outdated texts, and we varied whether the outdated, the updated, or no information was illustrated. In all experiments, we measured reading times for a target sentence referring to the updated information and the sentence following the target sentence. Results showed that target sentences’ reading times were faster for illustrated than for non-illustrated texts (Experiments 1 and 2). Moreover, reading times were longer when the outdated information was illustrated than when the updated information was illustrated (Experiment 3). These results suggest that pictures overall facilitate cognitive processes during reading, but their content matters: Pictures showing the updated information had a greater impact on reading times than pictures showing the outdated information. The results extend existing theories on knowledge revision but also reading comprehension, by demonstrating how pictures might influence cognitive processes during reading.

Reading and learning require not only the incorporation of new information but also the outdating and revision of information that is not or no longer accurate. Findings from several studies suggest that this process of knowledge revision is rather challenging, as outdated information continues to be automatically reactivated. The reactivated but outdated information can hinder comprehension processes (Johnson & Seifert, 1994; Lewandowsky et al., 2012; O’Brien et al., 1998, 2010; Rich & Zaragoza, 2016). This effect is also known as the continued influence effect (Ecker et al., 2022). Previous work has shown that explanations that include causal inferences about why the outdated information is no longer accurate are sufficient to reduce its reactivation and therefore facilitate the knowledge revision process (Johnson & Seifert, 1994; Kendeou et al., 2013, 2019).

Existing work has primarily used non-illustrated texts to evaluate revision processes and outcomes. However, most texts in textbooks, on the web, and in social media are multimodal. We posit that one factor that might facilitate knowledge revision processes is the illustration of the updated information via pictures. As pictures often positively affect learning outcomes (e.g., Mayer, 2021), it is important to investigate how pictures possibly influence knowledge revision processes. Understanding the extent to which and how pictures influence knowledge revision processes is essential for at least two reasons. First, knowledge revision is a central component of learning. By understanding which factors influence this process, it is possible to improve learning (i.e., instruction and instructional materials). Second, such understanding can expand and refine existing theories on knowledge revision during reading, such as the knowledge revision framework (Kendeou & O’Brien, 2014), by identifying how processing of pictures possibly contribute to knowledge revision.

In a series of three experiments, we aimed to identify how and to what extent pictures influence the knowledge revision process. On the one hand, we expected that illustrating updated information would strengthen the activation of the updated information and therefore facilitate knowledge revision processes (Experiments 1, 2, and 3). On the other hand, we expected that illustrating outdated information would strengthen the activation of outdated information and therefore hinder knowledge revision processes (Experiment 3). These hypotheses were based on studies showing better memory for illustrated text information than for non-illustrated text information (Levie & Lentz, 1982; Mayer, 2021). This set of experiments can help us understand under what conditions pictures can facilitate or hinder revision processes during reading. As a starting point in building the theoretical basis of this work, we will focus on the automatic activation process during the reading of non-illustrated texts.

## Activation and knowledge revision processes when reading non-illustrated texts

According to most text comprehension models, activation of prior knowledge (i.e., information presented in the text or general world knowledge) is necessary for comprehension (McNamara & Magliano, 2009). Many memory-based comprehension models assume an automatic activation process during reading (Albrecht & O’Brien, 1993; Kintsch, 1998; Myers & O’Brien, 1998; Sanford & Garrod, 2005). For example, the resonance-integration-validation (RI-Val; O’Brien & Cook, 2016a) model incorporates activation as the first of three automatic cognitive processes during reading. According to the RI-Val model, the activation process works with the resonance principle. That means that just-read information sends a signal to long-term memory and automatically activates related information (Myers & O’Brien, 1998; O’Brien & Cook, 2016a). Therefore, irrelevant, false, or outdated information may be automatically activated if it overlaps with the currently read content. The activated content influences subsequent comprehension processes if the activation is above a specific threshold. According to the RI-Val model, the activated information is then automatically integrated with the just-read content and validated (i.e., checked whether the integrated information is consistent; O’Brien & Cook, 2016a, 2016b).

Empirical evidence for the automatic activation of irrelevant or outdated information stems, for example, from research using an adapted version of the contradiction paradigm (O’Brien et al., 1998, 2010). In this paradigm, participants read one of three possible text conditions: consistent, inconsistent, or outdated. The focus is on measuring reading times for a target sentence (that is either consistent or inconsistent with preceding text) and the sentence following this target sentence (i.e., spillover sentence). The target and spillover sentences are the same in all conditions, but the information presented beforehand in the texts varies. For example, consider a text about Susan who loves an old oak tree in her front yard. The target sentence refers to the fact that this tree had to be cut down: “All that remained of the tree was a stump.” In the consistent condition, the readers learn that there was a huge thunderstorm, the tree was struck by lightning, and it had to be cut down. This information is consistent with the target sentence. In the inconsistent condition, Susan’s husband wants to cut down the tree, but Susan convinces him not to cut it down. This information is inconsistent with the target sentence. In the outdated condition, the same information as the inconsistent condition is presented but with one qualifying sentence. In this example, the qualifying sentence describes that the tree had to be cut down due to a thunderstorm. Thus, the initial information is not consistent with the target sentence, but with the addition of the qualifying sentence it is. After a background section, which is the same for all conditions, the target sentence is presented.

Reading times for this target sentence are typically the fastest in the consistent condition, followed by the outdated condition, and the slowest in the inconsistent condition. This difference “spills over” to the following sentence, the spillover sentence, which continues the story but does not directly mention consistent or inconsistent information (O’Brien et al., 2010). The difference in reading times between the consistent and the inconsistent conditions suggests that the earlier information about the tree is activated, integrated, and validated when reading the target sentence. More importantly, the reading times for the target and spillover sentences are significantly longer in the outdated condition than in the consistent condition—even though the target sentence is consistent with information presented in the consistent *and* the outdated condition (in both conditions, the tree must be cut down). This difference in reading times suggests that the outdated information (Susan convincing her husband not to cut down the tree) was activated, resulting in longer reading times than in the consistent condition.

Note that the difference in reading time between the outdated and the consistent condition is not necessarily an indicator that readers failed to update their knowledge. In fact, many participants can correctly answer that the tree was cut down. Thus, the reading time measure does not measure comprehension or knowledge updating directly. Instead, reading times show that outdated information affects comprehension and reasoning processes, even though participants have updated their knowledge (Ecker et al., 2015; Lewandowsky et al., 2012; Rich & Zaragoza, 2020). However, there must be some limits to the reactivation and influence of outdated information. Otherwise, revision of outdated information would never be possible.

The knowledge revision components framework (KReC; Kendeou & O’Brien, 2014) is one theoretical framework identifying the necessary cognitive processes for knowledge revision during reading. Consistent with the empirical findings described above, this framework assumes that once knowledge is encoded, it cannot be erased entirely from long-term memory and may be automatically activated when related information is presented. It further identifies three necessary cognitive processes for knowledge revision: co-activation, integration, and competing activation. First, the outdated and the updated information must be simultaneously activated and then integrated. That means the outdated and the updated information are connected in memory. According to the competing activation principle, revision is facilitated to the extent that the activation is drawn away from the outdated information towards the updated information. Note that there are also other theoretical assumptions about how and when knowledge revision occurs (Ayers & Reder, 1998; Lee et al., 2017; St. Jacques et al., 2013). In addition, knowledge revision or updating does not necessarily mean that only new, correct information is added to memory. For example, studies on the so-called misinformation effect suggest that later given but wrong information can distort event memories (Loftus, 1975; Loftus & Palmer, 1974). However, in the current experiments, we focus on cases in which one piece of information is given and later clearly revealed to be no longer true.

Empirical studies have so far often focused on how textual features influence the competing activation proposed within the KReC framework and, thus, knowledge revision (Cook & Guéraud, 2005; Ecker et al., 2015; Guéraud et al., 2018; Johnson & Seifert, 1994; Kendeou et al., 2013, 2019; Kendeou & O’Brien, 2014). For example, in a series of experiments, Kendeou et al. (2013) used a version of the adapted contradiction paradigm described above. When giving causal explanations as to why the previously provided information was outdated, reading times for the target sentences did not significantly differ between the consistent and the outdated condition. This indicates that the outdated information no longer interfered with the reading processes. Causal explanations seem especially beneficial in drawing away activation from outdated information, as they provide a rich elaboration network. This rich representational network for updated information strengthens the activation of the updated information, while drawing away activation from the outdated information. Above and beyond textual features (such as causal explanations), other factors may also influence whether outdated information is activated (Ecker et al., 2022), including accompanied pictures or illustrations.

## How pictures might influence the knowledge revision process

Findings from a wealth of research show that illustrated texts result in higher memory and comprehension than non-illustrated texts (Carney & Levin, 2002; Levie & Lentz, 1982; Mayer, 2021; Schnotz, 2014). Several mechanisms might contribute to this effect. First, pictures might guide attention toward the illustrated content (see Danielson et al., 2016, for a similar argument), increasing later memory for the illustrated information. Second, pictures are more similar to the outcome of comprehension—namely, a mental model—than text (Glenberg & Langston, 1992; Gyselinck & Tardieu, 1999). For example, Gyselinck and Tardieu (1999) argue, that “pictures can be viewed as one possible expression of a mental model, and presenting pictures may facilitate the construction of a mental model” (p. 212). Third, pictures are often easier to process than text. Studies on scene perception show that as little as 300 ms are sufficient to extract gist form a scene (Biederman et al., 1974; Henderson & Hollingworth, 1999). This ease in processing can lead to the subjective impression that illustrated information is more reliable/true than non-illustrated information (Newman et al., 2012). However, the “truthiness effect of pictures” has a rather small effect size and depends on additional conditions (Nadarevic et al., 2020; Newman et al., 2020).

Finally, following the cognitive theory of multimedia learning (CTML), having information presented in two different modalities (i.e., text and picture) can increase learning outcomes (Mayer, 2021). CTML theorizes that text and pictures are processed in two different, capacity-limited channels. When processing illustrated texts, learners first select the relevant text and picture information, construct a verbal and a pictorial mental model, and subsequently integrate both models into an integrated mental model. CTML argues that having information available in two different codes supports memory (Paivio, 1990) and increases the overall available processing capacity as information is processed in both channels. Moreover, it assumes that the integration of the verbal and the pictorial mental model is essential for deeper understanding.

Taken together, findings from this work suggest that illustrating a text with pictures can guide attention toward the depicted content, facilitate mental model construction, create the impression that the presented information is true, lead to more processing capacity available, and result in an integrated mental representation. All these factors might contribute to the construction of a more coherent and stable mental model in illustrated than in non-illustrated text. In turn, the more coherent and stable model can increase memory and comprehension.

When linking this beneficial effect of pictures with the competing activation principle of the KReC framework, one can argue that pictures might strengthen the activation of the content they illustrate. Consider the aforementioned text example about Susan. In the outdated condition, participants first learned that Susan decided to *not* cut down the oak tree. However, later it was cut down due to a thunderstorm. Illustrating the updated information with a picture of the fallen tree might result in a more coherent and stable mental model about the fallen oak tree. This might draw activation towards the updated information (fallen tree) and away from the outdated information (tree standing). When reading the target sentence about the fallen tree, the illustrated, updated information might be more activated than the non-illustrated, outdated information. Vice versa, illustrating the outdated information with a picture of the oak tree standing in the garden might lead to easier and faster activation of the outdated information than the updated information when reading the target sentence. Taken together, we expect the picture to influence the competing activation of outdated and updated information.

It is important to note, though, that previous studies investigating how pictures specifically influence knowledge revision have produced mixed results (Danielson et al., 2016; Mason et al., 2017). These studies used different combinations of refutational texts + graph (Danielson et al., 2016) or refutational texts + refutational graph (Mason et al., 2017). Refutational texts are texts designed in accordance with the KReC framework: First, the updated information *and* the incorrect/outdated information are described (co-activation and integration principle). Then, the correct explanation is given (competing activation principle). After participants had read illustrated or non-illustrated texts, knowledge revision was measured at several time points. In both studies, the graph did not influence knowledge revision above and beyond the effects of the refutational texts. However, none of the studies directly compared a standard text condition with a standard text + standard graph condition. Moreover, both studies measured knowledge revision via post-test knowledge scores. It is unclear if, and if so how, pictures directly influence cognitive processes while processing of the illustrated text. Other measures, such as reading times, can more directly assess these ongoing cognitive processes. In turn, understanding these processes can help to determine if and under what conditions a picture might influence the activation of outdated and updated information and therefore knowledge revision processes.

## The current experiments

In the current experiments, we investigated how pictures influence the activation of outdated and updated information. All three experiments used an adapted version of the contradiction paradigm described above. Within this paradigm, reading times are measured, which allows for measuring the ongoing cognitive processes more directly. In Experiments 1 and 2, we focused on whether illustrating the updated information would strengthen the activation of the updated information and therefore facilitate knowledge revision processes. In Experiment 3, we additionally focused on whether illustrating the outdated information would strengthen the activation of the outdated information and therefore hinder knowledge revision processes.

Specifically, in Experiment 1, participants read a text containing only consistent, updated information (consistent text condition) or a text containing outdated information that was later updated (outdated text condition). We varied whether the updated information was illustrated by a picture. Experiment 2 replicated Experiment 1 with a refined material set. In Experiment 3, participants read only the outdated texts. We varied whether the updated *or* the outdated information was illustrated by a picture. All experiments were preregistered at aspredicted.org and approved by the local ethic commission.

## Experiment 1

In Experiment 1, our aim was to examine if illustrated updated information would strengthen the activation of the updated information. We used the contradiction paradigm and relied on texts already used by O’Brien et al. (2010).^1^ This made it possible to compare the results with those in the extant literature. We varied the factor text condition (consistent information versus outdated information that is then updated) and picture (text is not illustrated vs. text is illustrated with picture showing the updated information). In line with the competing activation principle during knowledge revision (Kendeou & O’Brien, 2014) and research showing better comprehension and memory for illustrated texts compared with non-illustrated texts (e.g., Mayer, 2021), we hypothesized that in the outdated condition the picture would lead to faster reading times for target sentences compared with texts without pictures. We assumed that when contradictory information is available (i.e., outdated and updated information), illustrating the updated information would strengthen the activation of this information (competing activation principle). In the consistent condition, we did not expect any effect of the picture factor. This was due to two reasons. First, from a theoretical perspective we expected the picture to influence the competing activation of outdated and updated information. However, there is no outdated information in the consistent condition, and therefore no competing activation. Second, in a previous study we compared consistent illustrated and non-illustrated texts and did not find any effect of the picture on target sentences’ reading times (Frick & Schüler, 2023) indicating that with consistent texts, pictures have no add-on effect. To summarize this, we hypothesized a significant interaction effect between the factors text condition (consistent vs. outdated) and picture (non-illustrated vs. illustrated with a picture showing the updated information). For consistent texts, we expected no difference in the reading times for the target sentences as a function of the factor picture. For outdated texts, we expected faster reading times for the target sentences if the updated information was illustrated compared with if the updated information was not illustrated. The preregistration can be found online (https://aspredicted.org/ws89r.pdf).

### Method

#### Participants and design

We varied the factors text condition (consistent vs. outdated) and picture (non-illustrated vs. illustrated with a picture showing the updated information) within participants (i.e., 2 × 2 within-subjects design). We conducted a power simulation based on the data by O’Brien et al. (2010) and previous data (Frick & Schüler, 2023). Both factors were dummy-coded (consistent text = 0, outdated text = 1; non-illustrated = 0, illustrated = 1). The power simulation revealed that 400 participants were necessary to detect an interaction effect between the factors text condition and picture of −120 ms with sufficient power (1 − β >/= 0.8).^2^ Due to possible dropout and in line with our pre-registration, we recruited 450 participants. The participants were recruited via Prolific and received ₤4.25 for their participation. Ten participants had to be excluded due to data-saving issues or self-reported technical problems, three were older than 35 or did not report their age, and 16 answered more than 40% of the attention check tasks incorrectly (see below for details). Hence, 421 participants remained for the analysis (257 = male, 154 = female, 10 = non-binary, *M*_age_ = 28.23 years, *SD* = 4.56).

#### Materials

Sixteen texts were adapted from O’Brien et al. (2010) and slightly modified for our research purpose. Each text was presented in four conditions: consistent non-illustrated, consistent illustrated, outdated non-illustrated, and outdated illustrated. Each text consisted of six parts: an introduction, an elaboration section, a background section, the target sentence, the spillover sentence, and an ending. In the introduction (*M* = 10.06 words, *SD* = 1.82), a protagonist was introduced. The elaboration section contained the experimental manipulation (i.e., consistent information vs. outdated information, which was later updated) and, hence, differed between the consistent and the outdated version. In the original material, the elaboration sections differed in length. We adjusted the length so that the consistent (*M* = 95.94 words, *SD* = 3.78) and outdated (*M* = 95.63 words, *SD* = 2.69) text conditions were equal in length. The next section, the background section (*M* = 59.31 words, *SD* = 2.64), gave additional information about the protagonist but was irrelevant for the target sentence. It served to background the information given in the elaboration section making activation processes necessary when reading the target sentence. The target sentence referred to the information given in the elaboration section. The target sentences were controlled for length at character level (*M* = 40.19 characters, *SD* = 1.59). Then, the spillover sentence followed. The spillover sentences were controlled for length at character level as well (*M* = 38.43 characters, *SD* = 3.41). The ending concluded the story (*M* = 11.56 words, *SD* = 1.46).

In the conditions with pictures, the relevant (updated) information for the target sentence (e.g., the fallen tree) was illustrated by a picture (elaboration picture). This information was always given in the last sentence of the elaboration section. In addition, two other pictures were presented for each text to mask the purpose of the investigation: one picture illustrating a sentence of the introduction (introduction picture), and the other picture illustrating a sentence in the background section (background picture). The three pictures were colored, drawn by the research team, and depicted the scene described in the corresponding sentence. They were the same for the consistent and the outdated text condition. Between the last illustrated sentence and the target sentence were always at least two sentences. A full example text including the picture can be seen in Table 1.

**Table 1:**
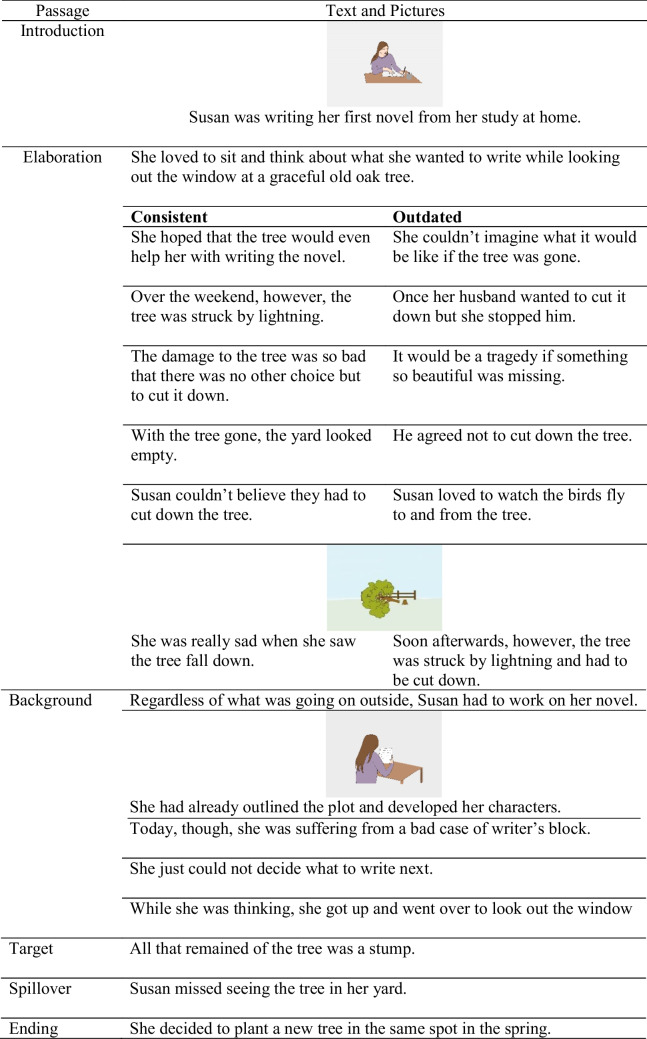
Example text and picture material for Experiment 1

Text is adapted from O’Brien et al. (2010). The texts were presented sentence-by-sentence. In the illustrated conditions, the picture was printed above the corresponding sentence.

To counterbalance texts and text conditions across participants, four lists were created that included 16 texts each. Every list contained each text only once and four texts in each of the four possible conditions. Participants were assigned to one of these four lists. The order in which the texts were presented within a list was randomized.

#### Measures

##### Dependent measure

We measured the reading time for the target sentences in milliseconds, estimated from their appearance on the screen until the space bar was pressed. For additional analysis, we also measured the reading times for spillover sentences. This and the following experiments were programmed in PsychoPy (Bridges et al., 2020; Peirce et al., 2019).

##### Attention-check measures

After each text, participants had to answer a short yes–no question about the text’s content. This text attention check measure ensured that participants were paying attention to the presented content. The questions did not refer to information given in the elaboration sections. After reading all 16 texts, participants also completed a picture attention check measure. Participants were asked to indicate whether they had seen the picture presented on the screen before (i.e., old) or whether it was new. We included eight old and eight new pictures in the task. The elaboration pictures were never included in this task. Importantly, participants were not informed beforehand about this attention check to avoid drawing their attention to the pictures and altering naturalistic processing. Therefore, participants only learned that they had to complete this picture attention check measure after reading all experimental texts.

#### Procedure

Participants were randomly assigned to one of the four lists. After answering demographic questions about age, gender, and educational level, participants learned that they were to read several short texts and answer one yes–no question about each text after its presentation. Each text started with the words “READY? Please press the space bar to start the story.” The texts were then presented sentence by sentence. Every time participants pressed the space bar, the current sentence (and picture, if applicable) disappeared, and the next sentence appeared. If a picture was present, it was printed above the corresponding sentence. After the final sentence of each text, the word “QUESTION” appeared on the screen for 1,000 ms. Then, the corresponding text attention check measure was presented. Participants responded via two different key presses: if the answer to the question was yes, participants pressed the K key; if it was no, they pressed the D key. To practice the whole procedure, participants read two consistent test text passages (one illustrated, one non-illustrated). Then, the 16 texts were presented in randomized order. After participants had read all 16 texts, they completed the picture attention check measure. Finally, participants answered questions about possible technical problems and were debriefed and thanked. The experiment lasted about 25 minutes.

### Results

The data were analyzed with R (R Core Team, 2022). Linear mixed-effect models were fitted to the data with the lme4 package (Bates et al., 2015); the lmerTest package (Kuznetsova et al., 2017) was used for the significance values. Single comparisons were computed with the emmeans package (Lenth, 2022).

The results of the text and picture attention-check measure can be seen in Table A1 in Appendix A. Furthermore, Table B1 in Appendix B lists the mean reading times for each illustrated sentence and the non-illustrated counterpart.

We excluded target sentences with exceptional fast (<500 ms) or slow (>7,000 ms) reading times, which resulted in 3.99% of removed data. Table 2 lists descriptive statistics.

**Table 2:** Mean reading times (in ms), standard deviations, estimated marginal means, and standard errors for the target sentences as a function of the factors text condition and picture

Text condition	Picture							
	Non-illustrated				Illustrated			
	*M*	*SD*	Marginal* M*	*SE*	*M*	*SD*	Marginal* M*	*SE*
Consistent	1,466.21	816.39	1,296.25	31.77	1,421.53	770.52	1,263.61	30.95
Outdated	1,505.89	846.85	1,325.57	32.49	1,446.94	758.16	1,284.10	31.47

Data distributions for this and the following experiments were graphically checked with q-q plots, scatter plots for residuals, and histograms. Because the reading time data were skewed, we applied a log-transformation.^3^

**Fig. 1:**
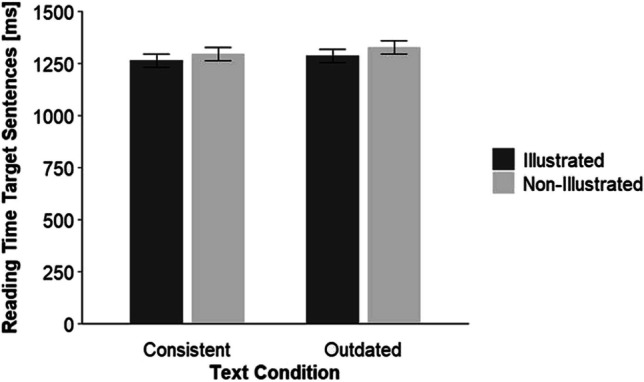
By the linear mixed-effect model estimated and back-transformed reading times as a function of the factors text condition and picture*. *Error bars show ±1 *SE*

We fitted a linear mixed-effect model with participants and texts (item) as random effects (random intercept) and text condition (dummy coded: consistent = 0 outdated = 1) and picture (dummy coded: non-illustrated = 0, illustrated = 1) as fixed effects to the reading times for the target sentences. Table 3 shows the model-estimated fixed and random effects. We found a significant main effect of the factor text condition, *p* = .028 with longer reading times for outdated than for consistent texts. Moreover, the factor picture, *p* = .012, was significant with longer reading times in the non-illustrated than in the illustrated conditions. The interaction between the factors text condition and picture was not significant, *p* = .662. Figure 1 illustrates this result.

**Table 3:** Fixed and random effects of the linear mixed-effects model for the target sentences

	Fixed effects				
	Estimate	*SE*	*df*	*t*	*p*
Intercept	7.17	0.02	71.51	292.39	<.001
Text condition^a^	0.02	0.01	6027	2.19	.028
Picture^b^	−0.03	0.01	6029	−2.51	.012
Text Condition × Picture	−0.01	0.01	6029	−0.44	.662
	Random effects				
	Variance	*SD*			
Participants^c^	0.12	0.35			
Items^c^	0.004	0.06			
Residual	0.08	0.29			

*N* target sentences = 6,467. *T* tests use Satterthwaite’s method.

^a^ dummy coded: consistent = 0, outdated = 1.

^b^ dummy coded: non-illustrated = 0, illustrated = 1.

^c^ random intercept.

In line with our preregistration, we also analysed the reading times for the spillover sentences (i.e., the sentence following the target sentence). We applied the same exclusion criteria as for the target sentences and additionally excluded reading times of spillover sentences that followed excluded target sentences (5.94% of removed data). The mean reading times for the spillover sentences as a function of the factors text condition and picture can be seen in Table C1 in Appendix C. We then log transformed the data and fitted a linear mixed-effect model to them. Again, participants and texts (item) were entered as random effects (random intercept), and text condition (dummy coded: consistent = 0, outdated = 1) and picture (dummy coded: non-illustrated = 0, illustrated = 1) were entered as fixed effects. The model revealed a significant main effect of the factor picture, *estimate* = −0.03, *SE* = 0.01, *t*(5905.98) = −2.99, *p* = .003. Reading times were significantly faster in illustrated conditions than in non-illustrated conditions. The effect of the factor text condition, *estimate* = 0.02, *SE* = 0.01, *t*(5904.72) = 1.46, *p* = .145, and the interaction effect, *estimate* = 0.02, *SE* = 0.01, *t*(5905.13) = 1.04, *p* = .299, were not significant.

## Discussion

In Experiment 1 we investigated whether pictures showing updated information can facilitate knowledge revision. To do so, we designed texts that either did or did not contain outdated information, which was later updated. Additionally, we varied whether the updated information was illustrated or not. We measured reading times for the target sentence referring to the elaboration section and for the spillover sentence. For outdated texts, we expected faster reading times for the target sentences in the illustrated than in the non-illustrated condition. For the consistent texts, we did not expect any effect of the factor picture.

We found a main effect of the factor picture with faster reading times in the illustrated than in the non-illustrated conditions in both text conditions. As hypothesized, reading times were faster in the outdated illustrated condition than in the outdated non-illustrated condition, suggesting the pictures might strengthen the activation of the updated information and thereby reduce the influence of outdated information. As the target sentences always referred to the updated information, the reduction in reading time could suggest that pictures did indeed strengthen the activation of the updated information. However, and contrary to our hypothesis, reading times were also faster in the consistent illustrated condition than in the consistent non-illustrated condition. This was especially surprising as an earlier study using a similar procedure did not find reduced reading times in the illustrated compared with the non-illustrated condition for consistent texts (Frick & Schüler, 2023). This finding suggests that illustrations overall facilitate reading comprehension processes.

Moreover, we also found a main effect of the factor text condition. Reading times on target sentences were longer for texts containing outdated information and therefore required knowledge revision compared with texts that only contained consistent information. This is in line with previous studies showing longer reading times for texts requiring knowledge revision (O’Brien et al., 2010).

As cognitive processes evoked by one sentence might also influence the reading time of the following sentence (i.e., spillover effect), we also analysed spillover sentences’ reading times. The spillover sentences’ reading times were faster for illustrated than for non-illustrated conditions. The text condition did not influence the spillover sentences’ reading times.

Taken together, we found faster reading times for target and spillover sentences for illustrated texts compared with non-illustrated texts for both text conditions. These findings only partially supported our hypothesis since we expected an effect of picture presentation *only* for the outdated text conditions. One possible explanation for this unexpected finding could be differential text-picture alignment between the two text conditions. More precisely, the same elaboration picture was combined with different sentences depending on the text condition (see, for example, the elaboration picture in Table 1). We conducted a follow-up experiment (*N* = 128) to explore whether the text-picture alignment differed depending on the text condition. It revealed that the sentence–picture combinations of the outdated text condition fitted significantly better than the sentence-picture combinations of the consistent text condition, *M*_Difference_ = −0.27, *t*(1887) = −7.03, 95% CI [−0.35, −0.20], *p* < .001. A more detailed description of this experiment can be found in the preregistration (https://aspredicted.org/79768.pdf) and on OSF (see link in open practices statement). To address this confounding variable, we revised the material set and conducted a replication in Experiment 2. This material set controlled better for text–picture alignment between the consistent and the outdated text conditions.

## Experiment 2

The aim of Experiment 2 was to replicate Experiment 1 with an improved material set. More precisely, the material set in Experiment 2 better controlled for text–picture alignment (see the Materials section for details). As in Experiment 1, we expected significantly faster reading times for target sentences in the outdated text illustrated condition compared with the outdated text non-illustrated condition. We did not expect any difference in reading times as a function of the factor picture for the consistent conditions. The preregistration can be found online (https://aspredicted.org/4fg69.pdf).

### Method

#### Participants and design

Again, we varied the factor text condition (consistent vs. outdated) and picture (non-illustrated vs. illustrated with picture showing the updated information) within participants (i.e., 2 × 2 within-subjects design). Based on the data of Experiment 1, we computed a power simulation. As in Experiment 1, both factors were dummy-coded (consistent text = 0, outdated text = 1; non-illustrated = 0, illustrated = 1). The power simulation revealed that 400 participants were necessary to detect an interaction effect between the factors text condition and picture of -85 ms with sufficient power (1 − β >/= 0.8).^4^ Due to possible drop out and in line with our preregistration, we recruited 450 participants. The participants were recruited via Prolific and received ₤4.25 for their participation. One participant withdrew the consent for data processing, for six participants data saving was not working, 10 participants were excluded due to self-reported technical problems, four were older than 35 or did not report their age, and seven participants made too many errors (error rate > 40%) in the attention check task. Hence, 422 participants remained (243 = male, 165 = female, 14 = non-binary, *M* = 28.56 *SD* = 4.53).

#### Materials

The materials consisted of eight texts adapted from Cook (2014, third Experiment),^5^ and eight newly generated texts (16 texts in total). The basic structure of the material was the same as in Experiment 1. Each text was presented in four conditions (i.e., consistent non-illustrated, consistent illustrated, outdated non-illustrated, outdated illustrated) and consisted of six different parts (i.e., an introduction, an elaboration section, a background section, the target sentence, the spillover sentence, and an ending). Again, in the illustrated conditions, the (updated) information conveyed through the last sentence of the elaboration section was illustrated by a colored picture drawn by the research team. Additionally, the first sentence of the elaboration and the second or third sentence of the background section were illustrated to mask the purpose of the investigation. In contrast to the texts used in Experiment 1, the consistent and the outdated elaboration section differed in only two sentences: The sentence of the elaboration section with the critical information differed in one word and one sentence was added at the end of the outdated elaboration section. For example, one text was about the protagonist Ellen, who buys new clothes. In the consistent condition, she sees a dress which she buys. In the outdated condition, she first sees an overall and shortly before buying it, she decides to buy a dress instead. In the outdated elaboration section, one qualifying sentence was added to describe this fact: “While walking through the store she found a dress which was way more beautiful.” Importantly, the sentence which was combined with the picture was the same for both conditions: “Ellen went to the checkout to buy the dress.” Hence, all pictures were presented with the same sentences in both conditions. This ensured that the text–picture alignment was the same in both conditions. The full example text, including the pictures, can be seen in Table 4. The counterbalancing of the different text versions was done as in Experiment 1.

**Table 4:**
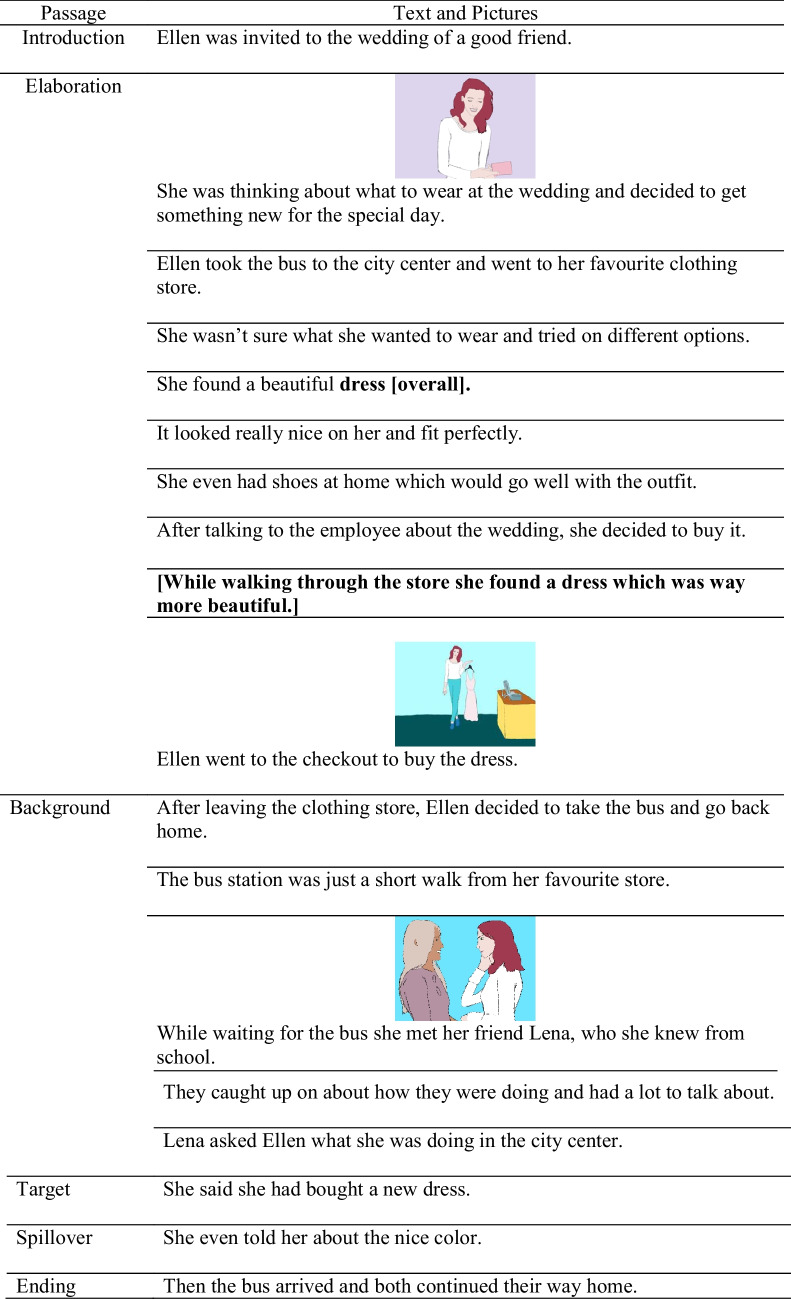
Example text and picture material for Experiment 2

The texts were presented sentence-by-sentence. In the illustrated conditions, the picture was printed above the corresponding sentence. Words in bold mark the difference between the outdated and the consistent conditions; words in brackets ([ ]) show the outdated text version.

#### Measures

Dependent measures and attention-check measures were the same as in Experiment 1.

#### Procedure

The procedure was the same as in Experiment 1.

### Results

The results of the text and picture attention-check measure can be seen in Table A1 in Appendix A. Furthermore, Table B1 in Appendix B lists the mean reading times for each illustrated sentence and the non-illustrated counterpart.

Again, we excluded target sentences with exceptional fast (<500 ms) or slow (>7,000 ms) reading times, which resulted in 5.20% of removed data. Table 5 lists the mean reading times and standard deviations for the target sentences as a function of the factors text condition and picture.

**Table 5:** Mean reading times (in ms), standard deviations, estimated marginal means, and standard errors for the target sentences as a function of the factors *text condition* and *picture*

Text condition	Picture							
	Non-illustrated				Illustrated			
	*M*	*SD*	Marginal* M*	*SE*	*M*	*SD*	Marginal* M*	*SE*
Consistent	1497.83	863.20	1314.56	40.22	1458.51	815.28	1288.76	39.44
Outdated	1544.36	887.62	1354.93	41.45	1487.05	867.85	1305.78	39.95

We log-transformed the reading time data as they were skewed. We fitted a linear mixed-effect model with participants and texts (item) as random effects (random intercept) and text condition (dummy coded: consistent = 0 outdated = 1) and picture (dummy coded: non-illustrated = 0, illustrated = 1) as fixed effects to the reading times for the target sentences. Table 6 shows the model-estimated fixed and random effects.

**Table 6:** Fixed and random effects of the linear mixed-effects model for the target sentences

	Fixed effects				
	Estimate	*SE*	*df*	*t*	*p*
Intercept	7.18	0.03	34.65	234.71	<.001
Text condition^a^	0.03	0.01	5,964.64	2.72	.007
Picture^b^	−0.02	0.01	5,966.73	−1.78	.076
Text Condition × Picture	−0.02	0.02	5,966.38	−1.09	.278
	Random effects				
	Variance	*SD*			
Participants^c^	0.12	0.34			
Items^c^	0.01	0.10			
Residual	0.10	0.32			

*N* target sentences = 6401. *T* tests use Satterthwaite’s method.

^a^ dummy coded: consistent = 0, outdated = 1.

^b^ dummy coded: non-illustrated = 0, illustrated = 1.

^c^ random intercept.

**Fig. 2:**
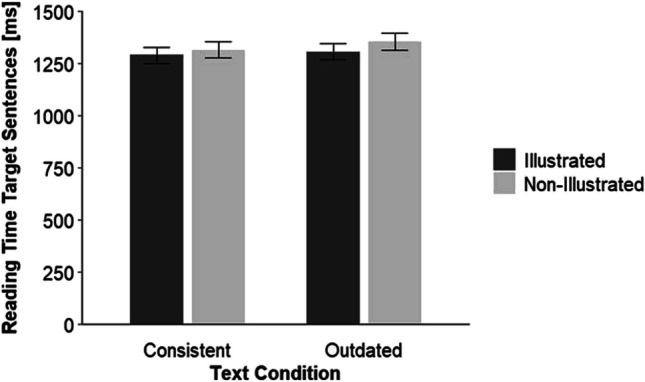
By the linear mixed-effect model estimated and back-transformed reading times as a function of the factors text condition and picture*. *Error bars show ±1 *SE*

This time, only the main effect of the factor text condition was significant, *p* = .007, with longer reading times for the outdated than for the consistent texts. The main effect of picture, *p* = .076, and the interaction effect of the factors text condition and picture, *p* = .278, were not significant. Figure 2 illustrates this result.

Again, we analysed the reading times for the spillover sentences to detect any delayed effects. After applying the same exclusion criteria as for the spillover sentences in Experiment 1 (6.87% of removed data), we log transformed the data, and fitted a linear mixed effect model to them. The descriptive reading times for the spillover sentences and the estimated marginal means can be seen in Table C2 in Appendix C. Again, participants and texts (item) were entered as random effects (random intercept), and text condition (dummy coded: consistent = 0 outdated = 1) and picture (dummy coded: non-illustrated = 0, illustrated = 1) were entered as fixed effects. The model revealed a significant main effect of the factor text condition, *estimate* = −0.04, *SE* = 0.01, *t*(5852) = 3.50, *p* < .001. Reading times were significantly faster in the consistent than the outdated condition. The effect of the factor picture, *estimate* = 0.004, *SE* = 0.01, *t*(5852) = 0.34, *p* = .731, and the interaction effect, *estimate* = −0.03, *SE* = 0.02, *t*(5852) = −1.77, *p* = .077, were not significant.

## Discussion

In Experiment 2, we replicated Experiment 1 with an improved material set. We found a main effect of the factor text condition for target and spillover sentences: target and spillover sentences’ reading times were significantly longer for texts containing outdated information compared with texts that only contained consistent information. For the target sentences’ reading times, this finding aligns with previous research (O’Brien et al., 2010) and with the results of Experiment 1.

More importantly, and contrary to our hypothesis, the interaction between the factors text condition and picture was not significant. Also, the main effect of picture, which was obtained in Experiment 1, was not significant. In Experiment 1, where a main effect of picture presentation was observed, pictures facilitated reading times independent of text condition. It is important to note that the descriptive data of Experiment 2 pointed towards faster reading times in the illustrated conditions as well. As no knowledge revision processes were necessary in the consistent condition, this might imply that pictures facilitate reading processes in general and not the specific cognitive processes involved in knowledge revision.

To summarize, in Experiments 1 and 2 we investigated whether illustrating the updated information with a picture would strengthen the activation of that information. The findings were relatively mixed and pointing into a potential general facilitative effect of pictures during reading. We reasoned that if pictures strengthen the activation of the depicted content in general, depicting outdated information should conversely strengthen the activation of the outdated information. We conducted Experiment 3 to explore this question.

## Experiment 3

In Experiments 1 and 2, we focused on comparing text conditions with pictures showing the updated information to text conditions without pictures. If pictures strengthen the activation of the depicted content, depicting outdated information should conversely strengthen the activation of the outdated information. In other words, if the outdated information is illustrated by a picture, knowledge revision might become even more challenging, as the outdated information is more likely to be reactivated. We conducted Experiment 3 to investigate this hypothesis. Participants were presented with the outdated text conditions used in Experiment 2, and we varied whether the text was illustrated by a picture showing the updated information, illustrated by a picture showing the outdated information, or non-illustrated. We expected longer reading times for the target and spillover sentences when the picture showed the outdated information compared with when the picture showed the updated information. We reasoned that if pictures facilitate activation of information, the difference between the condition with a picture showing the outdated information and the condition with a picture showing the updated information will be larger than the differences we found in Experiments 1 and 2, and ‘spill over’ to the next sentence. Moreover, we also expected longer reading times in the non-illustrated condition compared with the condition with a picture showing the updated information. The preregistration can be found online (https://aspredicted.org/4ka99.pdf).

### Method

#### Participants and design

We varied within participants the factor picture version: Text was illustrated by a picture showing the updated information, illustrated by a picture showing the outdated information, or non-illustrated. Based on the data from Experiments 1 and 2, we conducted a power simulation. The power simulation revealed that 420 participants were necessary to detect a difference of 70 ms between the two illustrated conditions (i.e., picture shows updated vs. outdated information) with sufficient power (1 − β >/= 0.8).^6^ Due to possible dropout and in line with our preregistration, we recruited 470 participants. The participants were recruited via Prolific and received ₤4.25 for their participation. Six participants were excluded due to self-reported technical problems, two reported only poor language skills, three did not report their age, and 18 participants made too many errors (error rate > 40%) in the attention check tasks. Hence, 441 participants remained (232 = male, 185 = female, 23 = non-binary, 1 = none of these options, *M*_age_ = 27.95 years, *SD* = 4.48).

#### Materials

We selected 12 outdated text conditions used in Experiment 2. We varied whether the texts were illustrated by a picture showing the updated information (same picture as in Experiment 2), illustrated by a picture showing the outdated information, or not illustrated. The pictures for the introduction and the background section were the same as in Experiment 2. Table 7 shows an example elaboration section with text and pictures. The picture showing the outdated information was similar to the picture showing the updated information, only the updated information was replaced by the outdated information (e.g., picture showing the protagonist with an overall vs. a dress). The counterbalancing of the different text versions was done as in Experiment 1. However, only three lists were generated this time, as only three different conditions existed.

**Table 7:**
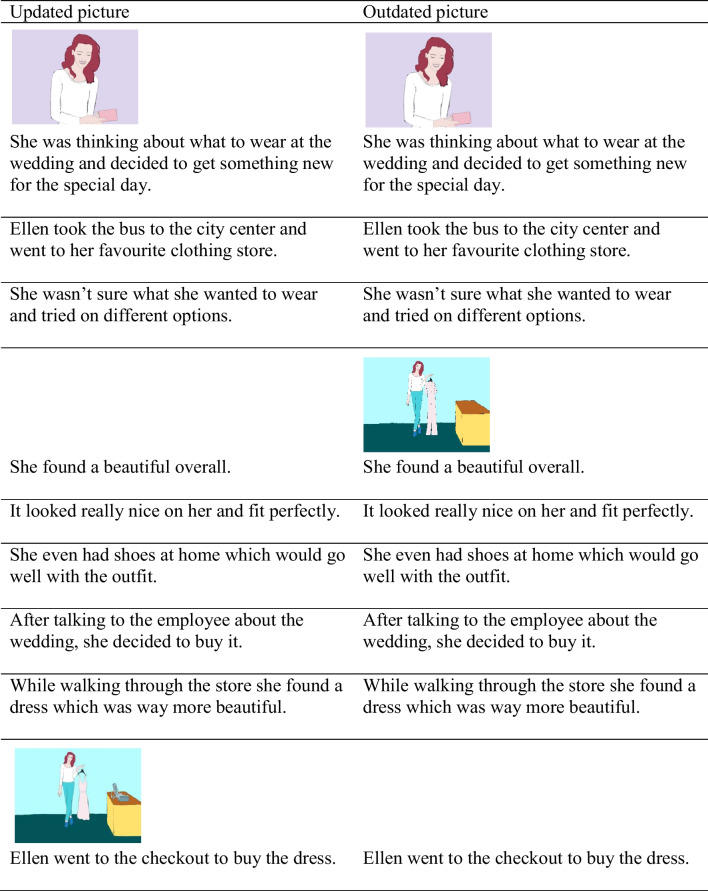
Example elaboration section for Experiment 3

In Experiment 3, only the outdated text version was used. The other texts parts were the same as in Experiment 2. The picture was printed above the corresponding sentence.

#### Measures

Dependent measures and attention-check measures were conceptually the same as in Experiment 1.

#### Procedure

The procedure was the same as in Experiment 1.

### Results

The results of the text and picture attention-check measure can be seen in Table A1 in Appendix A. Furthermore, Table B3 in Appendix B lists the mean reading times for each illustrated sentence and the non-illustrated counterpart.

We excluded target and spillover sentences with exceptional fast (<500 ms) or slow (>7,000 ms) reading times. Moreover, we excluded spillover sentences that followed excluded target sentences. This resulted in 2.60% of removed data for the target sentences and 4.34% of removed data for the spillover sentences. Table 8 lists the mean reading times and standard deviations for the target and spillover sentences as a function of the factor picture version and sentence type.

**Table 8:** Mean reading times (in ms), standard deviations, estimated marginal means, and standard errors as a function of the factors *picture version* and *sentence type*

Picture version	Sentence type							
	Target				Spillover			
	*M*	*SD*	Marginal* M*	*SE*	*M*	*SD*	Marginal* M*	*SE*
Non-illustrated	1,519.21	820.37	1,348.80	37.37	1,410.13	826.57	1,233.37	34.19
Updated	1,468.78	829.71	1,304.32	36.14	1,324.30	740.92	1,167.52	32.37
Outdated	1,499.24	852.57	1,325.84	36.73	1,343.53	727.29	1,192.70	33.07

We log transformed the reading time data as they were not normally distributed. We fitted a linear mixed-effect model with participants and text (item) as random effects (random intercepts) and picture version (dummy coded with updated picture as reference category) and sentence type (effect coded: 0.5 = target sentence, −0.5 = spillover sentence) as fixed effects to the log-transformed reading times.^7^ Table 9 (upper part) shows the model-estimated fixed and random effects.

**Table 9:** Model estimated fixed and random effects for the model with updated picture as reference category (upper part) and non-illustrated as reference category (lower part)

	Model 1 (updated picture = reference category)				
	Estimate	*SE*	*df*	*t*	*p*
Intercept	7.12	0.03	30.22	261.56	<.001
Outdated picture	0.02	0.01	9771	2.53	.011
Non-illustrated	0.04	0.01	9770	5.94	<.001
Sentence type^a^	0.11	0.01	9767	10.54	<.001
Outdated Picture × Sentence Type	−0.005	0.01	9767	−0.34	.738
Non-illustrated × Sentence Type	−0.02	0.01	9767	−1.44	.151
	Model 2 (non-illustrated = reference category)				
Intercept	7.16	0.03	30.22	263.18	<.001
Outdated picture	−0.03	0.01	9770	−3.41	<.001
Updated picture	−0.04	0.01	9770	−5.94	<.001
Sentence type^a^	0.09	0.01	9767	8.53	<.001
Outdated Picture × Sentence Type	0.02	0.01	9767	1.10	0.271
Updated Picture × Sentence Type	0.02	0.01	9767	1.44	0.151
	Random effects				
	Variance		*SD*		
Participants^b^	0.12		0.35		
Items^b^	0.01		0.07		
Residual	0.09		0.31		

*N* = 10,226 sentences (target and spillover). *T* tests use Satterthwaite’s method.

^a^ Effect coded: target sentence = 0.5, spillover sentence = −0.5.

^b^ Random intercept.

Consistent with our first hypothesis, we found significantly longer reading times when the outdated compared with when the updated information was illustrated, *p* = .011. Consistent with our second hypothesis, reading times were significantly longer in the non-illustrated condition than in the condition with a picture showing the updated information,* p* <. 001. There was no significant interaction between the picture version and sentence type.

**Fig. 3:**
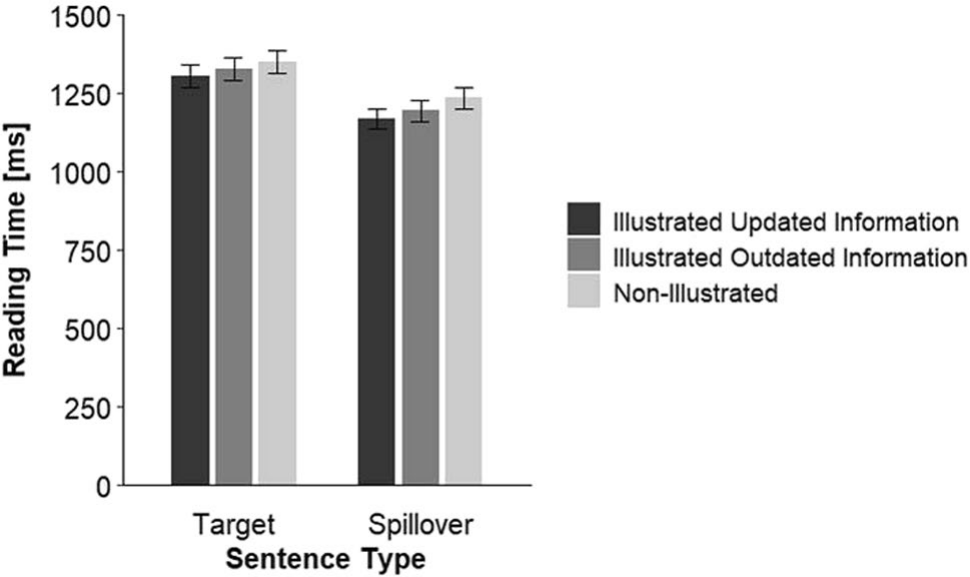
By the linear mixed-effect model estimated and back-transformed reading times as a function of the factors text version and sentence type*. *Error bars show ±1 *SE*

To compare the non-illustrated condition with the condition with a picture showing the outdated information, we fitted the same model as in the main analysis but with the non-illustrated condition as the reference category.^8^ Reading times were significantly longer in the non-illustrated condition than in the condition with a picture showing the outdated information,* p* <. 001. Figure 3 illustrates this result pattern.

### Discussion

In Experiment 3, we focused on examining further the effects of pictures on knowledge revision processes. We reasoned that if pictures strengthen the activation of the depicted content (as suggested by Experiments 1 and 2), depicting outdated information should conversely strengthen the activation of the outdated information. In other words, if the outdated information is illustrated by a picture, knowledge revision might become even more challenging, as the outdated information is more likely to be reactivated. Thus, we expected longer reading times for the target and spillover sentences when the picture showed the outdated information compared with when the picture showed the updated information. Additionally, we expected longer reading times in the non-illustrated condition compared with the condition with a picture showing the updated information. Results were in line with both hypotheses revealing longer reading times when the outdated compared with the updated information was illustrated and longer reading times in the non-illustrated compared with the updated illustrated condition. Surprisingly, reading times were also longer in the non-illustrated condition compared with the condition with a picture showing the outdated information.

Taken together, reading times were the longest in the non-illustrated condition, followed by the illustrated condition with a picture showing the outdated information, and the fastest in the illustrated condition with a picture showing the updated information. This might suggest that even pictures showing the outdated information can facilitate reading processes. Therefore, our data speak against the claim that illustrating outdated information with a picture hinders knowledge revision processes. However, this finding will need to be replicated in future studies to better understand its mechanisms.

Several mechanisms might explain why even outdated pictures seemed to facilitate knowledge revision processes during reading. One possibility is that pictures might draw attention to the illustrated content. This attention allocation is likely not only directed to the specific content (e.g., a dress or an overall) but also to the general concept (e.g., clothes). In the example presented in Table 7, this would mean that attention shifted towards the concept of clothes more generally. When reading the target sentence, all items connected to the concept of clothes might be activated more easily, leading to faster reading times. Nonetheless, the content of the pictures seems to be crucial, as reading times were faster when the updated than when the outdated information was illustrated.

## General discussion

Outdated information (i.e., information that is not or no longer accurate) continues to be automatically activated during reading (O’Brien et al., 2010). This can hinder further reading and comprehension processes (Johnson & Seifert, 1994; Lewandowsky et al., 2012; O’Brien et al., 1998, 2010; Rich & Zaragoza, 2016). Existing research mainly focused on non-illustrated texts and on how textual features influence the activation of outdated information (Kendeou et al., 2013; Tippett, 2010). However, as most texts are multimodal, it is important to understand how pictures influence the activation of outdated as well as updated information, and, hence, knowledge revision processes.

We situated this work within the KReC framework (Kendeou & O’Brien, 2014), which posits that knowledge revision takes place if the activation of the updated information is stronger than the activation of the outdated information (via a competing activation mechanism). We argued that pictures possibly strengthen the activation of the information they illustrate (e.g., Gyselinck & Tardieu, 1999; Mayer, 2021). Illustrating the updated information should consequently lead to stronger activation of the updated information compared with the outdated information, supporting knowledge revision. Vice versa, illustrating the outdated information should lead to stronger activation of the outdated information compared with the updated information, hindering knowledge revision.

We conducted a series of three experiments to test these hypotheses. All three experiments used an adapted version of the contradiction paradigm (O’Brien et al., 1998, 2010). In Experiments 1 and 2, participants read texts that contained outdated information that was later updated (i.e., outdated text condition) or texts that only contained the updated information (i.e., consistent text condition). We varied whether a picture illustrated the updated (consistent) information or not. In Experiment 3, participants read only the outdated text condition. We varied whether outdated, updated, or no information was illustrated. In all experiments, reading times for a target sentence referring to the elaboration section and the spillover sentence following the target sentence were measured.

Taken together, we found faster reading times for illustrated than for non-illustrated texts (Experiments 1 and 3; Experiment 2 descriptively). This finding demonstrates that pictures influence both reading and knowledge revision processes. We argued above that text information illustrated by a picture might yield a more coherent and stable mental model than non-illustrated text information (Gyselinck & Tardieu, 1999; Mayer, 2021). This more coherent and stable mental model may lead to a stronger activation of the illustrated information. Even though it would be tempting to conclude that pictures influence competing activation (principle proposed within the KReC framework), such a conclusion is not supported by the findings. The main effect of picture presentation on reading times and, more specifically, the faster reading times in the consistent illustrated conditions than in the consistent non-illustrated conditions (Experiment 1 and descriptively in Experiment 2) rather suggest that pictures might have influenced the activation principle in general, as no knowledge revision was required in the consistent text conditions. That is, pictures might generally facilitate the activation of related information. Furthermore, as the effect of the pictures did not depend on whether knowledge revision was required (i.e., there was no interaction between the text conditions and the picture in Experiments 1 and 2), it is also likely that pictures did not (only) influence the cognitive processes involved in knowledge revision but rather the cognitive processes involved in reading comprehension more broadly. This could also mean that pictures might not have (only) influenced activation processes, but the integration and validation processes proposed within the RI-Val model (O’Brien & Cook, 2016b) as well.

Next to this general effect of picture presentation on reading speed of the target sentence, we would like to note that reading times were faster when the updated than when the outdated information was illustrated (Experiment 3), suggesting that the content of the pictures seems to be crucial as well. If we assume that the picture presentation leads to a more stable and coherent mental model of the presented information (Gyselinck & Tardieu, 1999; Mayer, 2021), the mental model arising from illustrating outdated information should interfere more strongly with the reading of the target sentence than the mental model that arises when the updated information is illustrated. This could explain the difference between illustrating updated and outdated information in Experiment 3.

Finally, we also found longer reading times for outdated than for consistent texts (Experiments 1 and 2), which is in line with the encoding and activation principle proposed within the KReC framework (Kendeou & O’Brien, 2014) and theories of text comprehension (Kintsch, 1998; O’Brien & Cook, 2016a). As described above, once information is encoded in long-term memory, it is not entirely erased, even if it is outdated. Therefore, the outdated information can be activated through the automatic activation process. The longer reading times for the target sentences in the outdated conditions support that argument and suggest that the outdated information was indeed reactivated. This finding is robust and consistent with many other studies showing that outdated information is automatically activated (Guéraud et al., 2018; Kendeou et al., 2013; O’Brien et al., 1998, 2010).

## Limitations and future research

When interpreting these findings, it is important to note that we used rather simple texts and pictures about different main characters. The effect of pictures on knowledge revision processes may vary depending on the complexity of the material. It is, for example, possible that pictures may influence knowledge revision processes when the information to be revised is more complex. For example, a lot of research on the effect of illustrated versus non-illustrated texts focused on more complex mechanisms, such as weather phenomena (e.g., lightning formation, tornado formation) or the functioning of a pulley system (Eitel et al., 2013; Mayer, 2021; Schüler, 2019). It might be possible that the beneficial effect of pictures is stronger if the material depicted in the picture is more complex to describe in the text.

Moreover, our main dependent variable was reading times. While this variable can hint at the underlying cognitive processes and show whether the ongoing cognitive processes were facilitated or disrupted, it cannot answer the question of how exactly the picture changed the involved cognitive processes. For example, the reading time measure cannot determine which of the involved cognitive processes (i.e., activation, co-activation, but also integration and validation) were affected by the pictures. More detailed measures, such as eye-tracking or think-alouds, could provide further insights into these questions. Moreover, reading times do not directly measure comprehension, nor do they measure whether participants revised their knowledge. More direct measures, such as knowledge scores or reasoning tasks are necessary to access knowledge revision more directly. Future studies should also take these variables into account. Nevertheless, measuring the underlying cognitive processes via reading times is important in understanding whether pictures can influence knowledge revision processes.

Finally, future studies should also consider memory theories stemming from different but interconnected research areas such as neurobiological theories investigating memory effects. For example, studies using fMRI can provide insights into ongoing neuronal mechanisms (e.g., St. Jacques et al., 2013). Furthermore, studies differentiating between the continued influence effect (investigated in the current experiments) and the misinformation effect (i.e., later given, suggestive information distorts event memories; Loftus, 1975) might offer a deeper understanding for the involved cognitive processes and help us understand better when memories are updated (Ecker et al., 2015). By connecting these different research areas, it might be possible to understand further the exact processes involved in knowledge revision and updating.

Open questions aside, our results provide important new insights into knowledge revision processes. We were able to show that pictures illustrating updated or outdated information influenced reading and knowledge revision processes. As most texts we encounter in education and our current information ecosystem are multimodal, it is essential to understand how pictures influence cognitive processing.

## Appendix A

**Table A1:** Mean number of correct solved items of the text and picture attention-check measures for Experiments 1–3

Attention check measure	Experiment 1		Experiment 2		Experiment 3	
	*N*	*M* (*SD*)	*N*	*M* (*SD*)	*N*	*M* (*SD*)
Text	16	15.16 (1.11)	15	13.51 (1.46)	12	10.95 (1.00)
Picture	16	13.47 (2.02)	16	13.88 (1.99)	12	10.56 (1.38)

*Note.* Table reports the data after the exclusion of participants. Note that in Experiment 2, one of the text attention check items had to be excluded based on participants’ comments: Participants reported in the open comment section that this question was formulated ambiguously and both answers (yes and no) could have been correct.

*N* = number of items.

## Appendix B

**Table B1:** Mean reading times and standard deviations (*SD*s) for each illustrated sentence and the non-illustrated counterparts for Experiment 1

Condition	Introduction	Elaboration	Background
	*M* (*SD*)	*M* (*SD*)	*M* (*SD*)
Consistent non-illustrated	3,339.29 (8,213.39)	2,074.83 (1,885.93)	2,391.78 (4,216.53)
Consistent illustrated	3,888.03 (15,943.66)	2,680.40 (3,951.63)	2,537.73 (1,903.31)
Outdated non-illustrated	3,179.24 (11,488.31)	2,929.77 (2,548.42)	2,389.96 (3,934.69)
Outdated illustrated	3,508.99 (8,334.54)	3,248.93 (2,324.61)	2,660.92 (2,642.67)

In the elaboration section, the illustrated sentences did differ from the non-illustrated sentences.

**Table B2:** Mean reading times and standard deviations (*SD*s) for each illustrated sentence and the non-illustrated counterparts for Experiment 2

Condition	Elaboration 1	Elaboration 2	Background
	*M* (*SD*)	*M* (*SD*)	*M* (*SD*)
Consistent non-illustrated	3,311.19 (4,454.94)	1,938.37 (1,832.66)	2,277.07 (2,435.32)
Consistent illustrated	4,096.38 (8,337.72)	2,564.93 (2,532.24)	2,931.43 (5,039.23)
Outdated non-illustrated	3,383.59 (4,766.89)	2,014.07 (2,359.69)	2,349.24 (2,831.41)
Outdated illustrated	3,695.23 (3,594.85)	2,652.72 (2,546.31)	2,710.33 (2,568.41)

Elaboration 1 = first picture presented in the elaboration section; Elaboration 2 = second picture presented in the elaboration section.

**Table B3:** Mean reading times and standard deviations (*SD*s) for each illustrated sentence and the non-illustrated counterparts for Experiment 3

Condition	Elaboration 1	Elaboration 2	Background
	*M* (*SD*)	*M* (*SD*)	*M* (*SD*)
Picture updated	3,854.02 (5,369.64)	2,665.99 (5,342.80)	2,583.01 (1,882.07)
Picture outdated	3,887.95 (5,269.38)	2,481.20 (2,934.83)	2,750.06 (2,343.84)
Non-illustrated	3,322.62 (3,649.97)	2,246.56 (2,376.60)	2,272.21 (1,945.70)

Elaboration 1 = first picture presented in the elaboration section; Elaboration 2 = second picture presented in the elaboration section.

## Appendix C

**Table C1:** Mean reading times (in ms), standard deviations, estimated marginal means, and standard errors for the spillover sentences as a function of the factors text condition and picture in Experiment 1

Text condition	Picture							
	Non-illustrated				Illustrated			
	*M*	*SD*	Marginal* M*	*SE*	*M*	*SD*	Marginal* M*	*SE*
Consistent	1,363.15	701.25	1,217.52	30.87	1,326.97	706.68	1,181.71	29.95
Outdated	1,396.15	772.05	1,235.47	31.33	1,372.57	744.67	1,216.90	30.85

**Table C2:** Mean reading times (in ms), standard deviations, estimated marginal means, and standard errors for the spillover sentences as a function of the factors text condition and picture in Experiment 2

Text condition	Picture							
	Non-illustrated				Illustrated			
	*M*	*SD*	Marginal* M*	*SE*	*M*	*SD*	Marginal* M*	*SE*
Consistent	1,348.85	760.58	1,181.26	36.07	1,356.93	785.71	1,185.72	36.23
Outdated	1,405.88	832.49	1,227.35	37.47	1,375.00	803.51	1,198.68	36.60

## Footnotes

1 We thank Prof. O’Brien for providing the materials.

2 The other factors were set as follows: fixed effects: *estimate* intercept = 1,900 ms, *estimate* text condition = 130 ms, *estimate* picture = −20 ms. *SD* of random effects: *SD* participant = 600, *SD* text condition = 250, *SD* residual = 850.

3 This log transformation was not preregistered for Experiment 1.

4 The other factors were set as follows: fixed effects: *estimate* intercept = 1,400, *estimate* text condition = 100, *estimate* picture = −40. *SD* of random effects: *SD* participant = 600, *SD* text condition = 150, *SD* residual = 600.

5 We thank Prof. Cook for providing the materials.

6 The other factors were set as follows: fixed effects: *estimate* intercept = 1,400, *estimate* illustrated with picture showing updated information vs. non-illustrated = 40, *SD* of random effects: *SD* participant = 600, *SD* text condition = 200, *SD* residual = 700.

7 Please note that we deviated from our preregistered model based on a reviewer’s recommendation. Instead of computing a model for the target sentences’ reading times and a separate model for the spillover sentences’ reading times, we have included the factor sentence type in the model. This allowed us to directly compare target and spillover sentences’ reading times.

8 This comparison was not preregistered and computed based on a reviewer's recommendation.

## Declarations

### Open practices statement

All experiments have been preregistered at aspredicted.org (see links in the corresponding sections). Materials, data sets, and analysis scripts for all experiments can be found on OSF (https://osf.io/5jq94/?view_only=c77d513a7f4d47fab0b0be72cbd0be99).

## References

[CR1] Albrecht, J. E., & O’Brien, E. J. (1993). Updating a mental model: Maintaining both local and global coherence. *Journal of Experimental Psychology: Learning, Memory, and Cognition,**19*(5), 1061–1070. 10.1037/0278-7393.19.5.1061

[CR2] Ayers, M. S., & Reder, L. M. (1998). A theoretical review of the misinformation effect: Predictions from an activation-based memory model. *Psychonomic Bulletin & Review,**5*(1), 1–21. 10.3758/BF03209454

[CR3] Bates, D., Mächler, M., Bolker, B., & Walker, S. (2015). Fitting linear mixed-effects models using lme4. *Journal of Statistical Software*, *67*(1), 1–48. 10.18637/jss.v067.i01

[CR4] Biederman, I., Rabinowitz, J. C., Glass, A. L., & Stacy, E. W. (1974). On the information extracted from a glance at a scene. *Journal of Experimental Psychology,**103*(3), 597–600. 10.1037/h0037158 4448962 10.1037/h0037158

[CR5] Bridges, D., Pitiot, A., MacAskill, M. R., & Peirce, J. W. (2020). The timing mega-study: Comparing range of experiment generators, both lab-based and online. *PeerJ*, *8*, Article e9414. 10.7717/peerj.9414 10.7717/peerj.9414 PMC7512138 33005482

[CR6] Carney, R. N., & Levin, J. R. (2002). Pictorial illustrations still improve students’ learning from text. *Educational Psychology Review,**14*(1), 5–26. 10.1023/A:1013176309260

[CR7] Cook, A. E. (2014). Processing anomalous anaphors. *Memory & Cognition,**42*(7), 1171–1185. 10.3758/s13421-014-0415-0 24796775 10.3758/s13421-014-0415-0

[CR8] Cook, A. E., & Guéraud, S. (2005). What have we been missing? The role of general world knowledge in discourse processing. *Discourse Processes,**39*(2/3), 265–278. 10.1080/0163853X.2005.9651683

[CR9] Danielson, R. W., Sinatra, G. M., & Kendeou, P. (2016). Augmenting the refutation text effect with analogies and graphics. *Discourse Processes,**53*(5/6), 392–414. 10.1080/0163853X.2016.1166334

[CR10] Ecker, U. K. H., Lewandowsky, S., Cheung, C. S. C., & Maybery, M. T. (2015). He did it! She did it! No, she did not! Multiple causal explanations and the continued influence of misinformation. *Journal of Memory and Language,**85*, 101–115. 10.1016/j.jml.2015.09.002

[CR11] Ecker, U. K. H., Lewandowsky, S., Cook, J., Schmid, P., Fazio, L. K., Brashier, N., Kendeou, P., Vraga, E. K., & Amazeen, M. A. (2022). The psychological drivers of misinformation belief and its resistance to correction. *Nature Reviews Psychology,**1*(1), 13–29. 10.1038/s44159-021-00006-y

[CR12] Eitel, A., Scheiter, K., Schüler, A., Nyström, M., & Holmqvist, K. (2013). How a picture facilitates the process of learning from text: Evidence for scaffolding. *Learning and Instruction,**28*, 48–63. 10.1016/j.learninstruc.2013.05.002

[CR13] Frick, P., & Schüler, A. (2023). Extending the theoretical foundations of multimedia learning: Activation, integration, and validation occur when processing illustrated texts. *Learning and Instruction*, *87*(June), Article 101800. 10.1016/j.learninstruc.2023.101800

[CR14] Glenberg, A. M., & Langston, W. E. (1992). Comprehension of illustrated text: Pictures help to build mental models. *Journal of Memory and Language,**31*(2), 129–151. 10.1016/0749-596X(92)90008-L

[CR15] Guéraud, S., Walsh, E. K., Cook, A. E., & O’Brien, E. J. (2018). Validating information during reading: The effect of recency. *Journal of Research in Reading,**41*, 85–101. 10.1111/1467-9817.12244

[CR16] Gyselinck, V., & Tardieu, H. (1999). The role of illustrations in text comprehension: What, when, for whom, and why? In H. van Oostendorp & S. R. Goldman (Eds.), *The construction of mental representations during reading* (pp. 195–218). Erlbaum. 10.4324/9781410603050

[CR17] Henderson, J. M., & Hollingworth, A. (1999). High-level scene perception. *Annual Review of Psychology,**50*(1), 243–271. 10.1146/annurev.psych.50.1.243 10074679 10.1146/annurev.psych.50.1.243

[CR18] Johnson, H. M., & Seifert, C. M. (1994). Sources of the continued influence effect: When misinformation in memory affects later inferences. *Journal of Experimental Psychology: Learning, Memory, and Cognition,**20*(6), 1420–1436. 10.1037/0278-7393.20.6.1420

[CR19] Kendeou, P., Butterfuss, R., Kim, J., & Van Boekel, M. (2019). Knowledge revision through the lenses of the three-pronged approach. *Memory & Cognition,**47*(1), 33–46. 10.3758/s13421-018-0848-y 30117115 10.3758/s13421-018-0848-y

[CR20] Kendeou, P., & O’Brien, E. J. (2014). The knowledge revision components (KReC) framework: Processes and mechanisms. In J. L. G. Braasch & D. N. Rapp (Eds.), *Processing inaccurate information: Theoretical and applied perspectives from cognitive science and the educational sciences* (pp. 353–377). Boston Review.

[CR21] Kendeou, P., Smith, E. R., & O’Brien, E. J. (2013). Updating during reading comprehension: Why causality matters. *Journal of Experimental Psychology: Learning, Memory, and Cognition,**39*(3), 854–865. 10.1037/a0029468 22845069 10.1037/a0029468

[CR22] Kintsch, W. (1998). *Comprehension: A paradigm for cognition*. Cambridge University Press.

[CR23] Kuznetsova, A., Brockhoff, P. B., & Christensen, R. H. B. (2017). lmerTest package: Tests in linear mixed effects models. *Journal of Statistical Software*, *82*(13), 1–26. 10.18637/jss.v082.i13

[CR24] Lee, J. L. C., Nader, K., & Schiller, D. (2017). An update on memory reconsolidation updating. *Trends in Cognitive Sciences,**21*(7), 531–545. 10.1016/j.tics.2017.04.006 28495311 10.1016/j.tics.2017.04.006 PMC5605913

[CR25] Lenth, R. V. (2022). *emmeans: Estimated marginal means, aka least-squares means* (R Package Version 1.8.3). https://cran.r-project.org/package=emmeans

[CR26] Levie, W. H., & Lentz, R. (1982). Effects of text illustrations: A review of research. *Educational Communication & Technology,**30*(4), 195–232.

[CR27] Lewandowsky, S., Ecker, U. K. H., Seifert, C. M., Schwarz, N., & Cook, J. (2012). Misinformation and its correction: Continued influence and successful debiasing. *Psychological Science in the Public Interest,**13*(3), 106–131. 10.1177/1529100612451018 26173286 10.1177/1529100612451018

[CR28] Loftus, E. F. (1975). Leading questions and the eyewitness report. *Cognitive Psychology,**7*(4), 560–572. 10.1016/0010-0285(75)90023-7

[CR29] Loftus, E. F., & Palmer, J. C. (1974). Reconstruction of automobile destruction: An example of the interaction between language and memory. *Journal of Verbal Learning and Verbal Behavior,**13*(5), 585–589. 10.1016/S0022-5371(74)80011-3

[CR30] Mason, L., Baldi, R., Di Ronco, S., Scrimin, S., Danielson, R. W., & Sinatra, G. M. (2017). Textual and graphical refutations: Effects on conceptual change learning. *Contemporary Educational Psychology,**49*, 275–288. 10.1016/j.cedpsych.2017.03.007

[CR31] Mayer, R. E. (2021). *Multimedia learning* (3rd ed.). Cambridge University Press.

[CR32] McNamara, D. S., & Magliano, J. (2009). Toward a comprehensive model of comprehension. In B. H. Ross (Ed.), *Psychology of learning and motivation: Advances in research and theory* (Vol. 51, pp. 297–384). 10.1016/S0079-7421(09)51009-2

[CR33] Myers, J. L., & O’Brien, E. J. (1998). Accessing the discourse representation during reading. *Discourse Processes,**26*(2/3), 131–157. 10.1080/01638539809545042

[CR34] Nadarevic, L., Reber, R., Helmecke, A. J., & Köse, D. (2020). Perceived truth of statements and simulated social media postings: an experimental investigation of source credibility, repeated exposure, and presentation format. *Cognitive Research: Principles and Implications*, *5*(1). 10.1186/s41235-020-00251-4 10.1186/s41235-020-00251-4 PMC7656226 33175284

[CR35] Newman, E. J., Garry, M., Bernstein, D. M., Kantner, J., & Lindsay, D. S. (2012). Nonprobative photographs (or words) inflate truthiness. *Psychonomic Bulletin & Review,**19*(5), 969–974. 10.3758/s13423-012-0292-0 22869334 10.3758/s13423-012-0292-0

[CR36] Newman, E. J., Jalbert, M. C., Schwarz, N., & Ly, D. P. (2020). Truthiness, the illusory truth effect, and the role of need for cognition. *Consciousness and Cognition*, *78*(May 2019), Article 102866. 10.1016/j.concog.2019.102866 10.1016/j.concog.2019.102866 31935624

[CR37] O’Brien, E. J., Albrecht, J. E., Rizzella, M. L., & Halleran, J. G. (1998). Updating a situation model: A memory-based text processing view. *Journal of Experimental Psychology: Learning Memory and Cognition,**24*(5), 1200–1210. 10.1037/0278-7393.24.5.1200 9747530 10.1037//0278-7393.24.5.1200

[CR38] O’Brien, E. J., & Cook, A. E. (2016a). Separating the activation, integration, and validation components of reading. In B. H. Ross (Ed.), *Psychology of learning and motivation* (Vol. 65, pp. 249–276). Academic Press. 10.1016/bs.plm.2016.03.004

[CR39] O’Brien, E. J., & Cook, A. E. (2016b). Coherence threshold and the continuity of processing: The RI-Val model of comprehension. *Discourse Processes,**53*(5/6), 326–338. 10.1080/0163853X.2015.1123341

[CR40] O’Brien, E. J., Cook, A. E., & Guéraud, S. (2010). Accessibility of outdated information. *Journal of Experimental Psychology: Learning, Memory, and Cognition,**36*(4), 979–991. 10.1037/a0019763 20565213 10.1037/a0019763

[CR41] Paivio, A. (1990). Mental representations: A dual coding approach. *Oxford University Press*. 10.1093/acprof:oso/9780195066661.001.0001

[CR42] Peirce, J., Gray, J. R., Simpson, S., MacAskill, M., Höchenberger, R., Sogo, H., Kastman, E., & Lindeløv, J. K. (2019). PsychoPy2: Experiments in behavior made easy. *Behavior Research Methods,**51*(1), 195–203. 10.3758/s13428-018-01193-y 30734206 10.3758/s13428-018-01193-y PMC6420413

[CR43] R Core Team. (2022). *R: A language and environment for statistical computing*

[CR44] Rich, P. R., & Zaragoza, M. S. (2016). The continued influence of implied and explicitly stated misinformation in news reports. *Journal of Experimental Psychology: Learning, Memory, and Cognition,**42*(1), 62–74. 10.1037/xlm0000155 26147670 10.1037/xlm0000155

[CR45] Rich, P. R., & Zaragoza, M. S. (2020). Correcting misinformation in news stories: An investigation of correction timing and correction durability. *Journal of Applied Research in Memory and Cognition,**9*(3), 310–322. 10.1016/j.jarmac.2020.04.001

[CR46] Sanford, A. J., & Garrod, S. C. (2005). Memory-based approaches and beyond. *Discourse Processes,**39*(2/3), 205–224. 10.1080/0163853X.2005.9651680

[CR47] Schnotz, W. (2014). Integrated model of text and picture comprehension. In R. E. Mayer (Ed.), *The Cambridge handbook of multimedia learning* (2nd ed., pp. 72–103). Cambridge University Press. 10.1017/CBO9781139547369.006

[CR48] Schüler, A. (2019). The integration of information in a digital, multi-modal learning environment. *Learning and Instruction,**59*, 76–87. 10.1016/j.learninstruc.2017.12.005

[CR49] St. Jacques, P. L., Olm, C., & Schacter, D. L. (2013). Neural mechanisms of reactivation-induced updating that enhance and distort memory. *Proceedings of the National Academy of Sciences,**110*(49), 19671–19678. 10.1073/pnas.1319630110 10.1073/pnas.1319630110 PMC3856820 24191059

[CR50] Tippett, C. D. (2010). Refutation text in science education: A review of two decades of research. *International Journal of Science and Mathematics Education,**8*(6), 951–970. 10.1007/s10763-010-9203-x

